# Combination regimen of granulocyte colony-stimulating factor and recombinant human thrombopoietin improves the curative effect on elderly patients with leukemia through inducing pyroptosis and ferroptosis of leukemia cells

**DOI:** 10.1038/s41417-022-00497-8

**Published:** 2022-06-29

**Authors:** Xiaobin Wang, Xiaoyu Liu, Huihan Wang

**Affiliations:** Department of Hematology, Shengjing Hospital, China Medical University, Shenyang, Liaoning 110035 China

**Keywords:** Chemotherapy, Chemotherapy

## Abstract

Leukemia ranks as the one of most common causes of death from tumor. 51.4% of patients with leukemia are over 65 years old. However, the median overall survival (OS) of elderly leukemia patients is less than one year. It is urgent to explore more effective treatments for elderly patients with leukemia. Our recent prospective phase II single-arm study has revealed that combination regimen of granulocyte colony-stimulating factor (G-CSF) and recombinant human thrombopoietin (rhTPO) could improve the curative effect on elderly patients with leukemia, yet the precise mechanism remains unknown. This study demonstrated that combination of G-CSF and rhTPO showed greater effect on suppressing leukemia growth than G-CSF or rhTPO alone in vitro and in vivo. Mechanistically, G-CSF induced pyroptosis through ELANE in leukemia cells. Besides, rhTPO triggered ferroptosis by EP300 in leukemia cells. Moreover, rhTPO suppressed glutathione peroxidase 4 (GPX4) expression to induce ferroptosis through blocking the interaction between EP300 and *GPX4* gene promoter via associating with EP300. In summary, this study illuminated that combination regimen of G-CSF and rhTPO improved the curative effect on elderly patients with leukemia through inducing pyroptosis and ferroptosis of leukemia cells. Therefore, our results provided a theoretical basis for combination regimen of G-CSF and rhTPO treating leukemia and potential therapeutic targets for leukemia.

## Introduction

Leukemia is a life-threatening disease inducing by malignant disorders of blood cells and bone marrow cells [1, 2], which ranks as the one of most common causes of death from tumor [3]. In 2017, 518,485 new cases and 347,583 deaths of leukemia occurred worldwide [3, 4]. Among patients with leukemia, 51.4% of them are over 65 years old [4]. With the aging of society, the proportion of elderly leukemia will increase year by year [3]. However, in the past 30 years, the 5-years survival rate of elderly leukemia patients over 60 years old is <20% due to the characteristics of elderly patients, such as numerous complications and severe bone marrow suppression after chemotherapy [5, 6]. More badly, the median OS of elderly leukemia patients is <1 year [7, 8]. Therefore, it is urgent to explore more effective treatments for elderly patients with leukemia.

Granulocyte colony-stimulating factor (G-CSF) is the first growth factor used for the treatment of leukemia in elderly patients, combined with low-dose cytarabine and aclarubicin (CAG regimen) [9]. Numerous studies have revealed that the use of G-CSF after chemotherapy reduces the neutropenia period, the risk of infection and mortality of patients [10, 11]. Besides, administration of G-CSF before or during chemotherapy enhances the cytotoxic effect of S-phase specific drugs (such as arabinoside) on leukemia cells through promoting cell cycle progression [12, 13]. However, the use of G-CSF remains controversial due to the increased risk of relapse and poor effect on neutropenic complications [14]. Thus, combination of G-CSF and other drugs might overcome limitations of G-CSF to improve curative effects of G-CSF on leukemia.

Thrombopoietin (TPO) is a critical cytokine that induces megakaryopoiesis and platelet production [15, 16]. Recombinant human thrombopoietin (rhTPO) is usually utilized for the treatment of thrombocytopenia after chemotherapy [17, 18]. To date, the effect of rhTPO in leukemia remains unclear. Our recent prospective phase II single-arm study has revealed that combination regimen of G-CSF and rhTPO is safer and improves the curative effect on elderly patients with acute myeloid leukemia (AML) [19]. However, the precise mechanism of combination regimen of G-CSF and rhTPO regulation of leukemia cells needs further exploration.

Therefore, the primary aim of this study was to investigate the mechanism how G-CSF and rhTPO treated leukemia using in vitro cell experiments and in vivo experiments in nude mice.

## Materials and methods

### Cell culture

Human leukemia cell lines including HL-60 and KG1a were purchased from the Cell Bank at the Chinese Academy of Sciences (Shanghai, China). Cells were cultured in the RPMI-1640 medium contained 10% calf fetal serum and antibiotics in an atmosphere with 5% CO_2_ at 37 °C.

### Cell treatments

Recombinant human G-CSF (10 ng/mL, #P00034, Solarbio, Shanghai, China) and rhTPO (1 ng/mL, #P00192, Solarbio) dissolved in phosphate buffered saline (PBS) were used to treat HL-60 and KG1a cells in this study. Besides, small interfering RNAs (siRNAs) or vectors were transfected into HL-60 cells using Lipofectamine 2000 (Thermo Fisher Scientific, Waltham, MA, USA). Then cells were collected for subsequent experiments (except CCK-8 assay) at 48 h after the transfection.

### Cell Counting Kit-8 (CCK-8) assay

CCK-8 assay was performed to detect the proliferation rates of HL-60 and KG1a cells. First, cells were seeded in the 96-well plate at the concentration of 1 × 10^4^/ml per well. 10 µL CCK-8 solution (Beyotime Biotechnology, Shanghai, China) at a 1/10 dilution was added into each well to incubate HL-60 and KG1a cells at 37 °C for 2 h after indicated transfections. Subsequently, absorbance at 450 nm was assayed by Multiscan MK3 (Thermo Fisher Scientific, Waltham, MA, USA). Next, the means of the optical density (OD) were used to calculate the proliferation rate using the below formula: (OD of treatment group/OD of control group) × 100 %.

### Flow cytometric analysis for programed cell death

HL-60 and KG1a cells from each group were collected and washed twice with incubation buffer including 10 mmol/L HEPES/NaOH (pH 7.4), 140 mmol/L NaCl and 5 mmol/L CaCl2. Next, Cells were resuspended into 100 μl PBS containing 1.5 μg/mL Annexin V and moderate Propidium iodide (PI) (Thermo Fisher Scientific) and incubated at room temperature (RT) for 15 min in dark. After washing by PBS three times, cells were resuspended by incubation buffer and analyzed by flow cytometry (FACSARIA, BD Biosciences, San Jose, CA, USA) as previously described [20, 21].

### In vivo assay

Four-week-old nude mice were raised in an environment free of pathogen at the experimental animal center. First, xenograft tumor growth models were established by subcutaneous injection of 1 × 10^7^ KG1a cells into the right dorsal flanks of nude mice. Then mice were randomized into four groups: control, G-CSF, rhTPO, G-CSF + rhTPO groups. Five mice were in each group. At 3 days after injection of KG1a cells, 45.05 μg/kg G-CSF and 225.25 μg/kg rhTPO dissolved in physiological saline were given by tail vein injection every 3 days. The same amount of physiological saline was injected into mice of control group. Subsequently, the size of xenograft tumors formed in nude mice was measured every 3 days. Then nude mice were sacrificed at day 21 after injection of KG1a cells followed by the measure of size and weight of xenograft tumors. All animal experiments were performed in accordance with the Ethic Committee of the China Medical University at Shengjing Hospital.

### Western Blot (WB)

WB was performed to detect cellular level of target proteins in HL-60 and KG1a cells. Briefly, equal amounts of protein (30 μg) from each group were loaded and separated by 10% SDS-polyacrylamide gel electrophoresis (SDS-PAGE) and subsequently transferred onto Immobilon-P membranes (Merck Millipore, Billerica, MA, USA). Subsequently, the membranes were blocked with 5% nonfat milk for 2 h at RT followed by the incubation with the primary antibodies in 1% nonfat milk overnight at 4 °C. After primary antibody incubation, the membranes were washed 4 times by Tris-buffered saline contained 0.1% Tween20 (TBST) and then exposed to secondary antibodies including peroxidase-Rabbit Anti-Goat IgG (1:30000, # BA1060, Boster, Wuhan, Hubei, China) and peroxidase-Rabbit Anti-Rat IgG (1:20000, # BA1058, Boster). The signals of targeted proteins were detected by the SuperSignal West Femto Maximum Sensitivity Substrate kit obtained from Thermo Fisher Scientific. Primary antibodies used in the present study were listed as follow: anti-ELANE (1:500, # bs-6982R, Bioss, Beijing, China), anti-cleaved N-terminal GSDMD (1:1000, #ab215203, Abcam, Cambridge, UK), anti-GAPDH (1:10000, #KC-5G5, Aksomics, Shanghai, China).

### ELISA

The levels of released IL-1β and IL-18 were measured by ELISA kits (#DG10307H and # DG10298H) obtained from Winter Song Boye (Beijing, China). Briefly, deionized water was used to dilute the washing buffer to 1× application buffer. Then 50 μL standards in different concentrations and 10 μL samples were added into different wells of 96-well plate. Triplicates were made for each sample. Next, 100 μL reagent labeled with enzyme was added into each well except the blank wells to incubate samples at 37 °C for 1 h. After washing, 50 μL color reagent A and B were added into each well to incubate samples at 37 °C for 15 min in dark followed by the termination of reaction using 50 μL termination solution. Finally, absorbance at 450 nm was immediately assayed by Multiscan MK3 (Thermo Fisher Scientific).

### Co-immunoprecipitation (Co-IP)

KG1a cells were lysed by the non-denaturing lysis buffer. Next, the supernatant of cell lysis was pre-cleaned with protein A/G magnetic beads (Thermo Fisher Scientific) for 2 h at 4 °C. Subsequently, about 300 μg of protein were incubated with 1μg G-CSF antibody (#ab181053, Abcam) or EP300 antibody (#ab275378, Abcam) and 25 microliters of protein A/G magnetic beads for immunoprecipitation at 4 °C overnight. Following the incubation with antibody and protein A/G magnetic beads, protein A/G magnetic beads were collected using magnetic separation device (Thermo Fisher Scientific), and precipitated complexes were cleansed by washing buffer (Thermo Fisher Scientific). Finally, bound proteins were analyzed by WB using anti-ELANE (1:500, # bs-6982R, Bioss) or anti-TPO (1:500, # ab196026, Abcam). Rabbit IgG was used for negative control.

### Detection of malondialdehyde (MDA)

First, HL-60 and KG1a cells were lysed with 1× ice-cold RIPA lysis buffer (Beyotime, Shanghai, China) and centrifuged followed by discarding deposit. Next, the supernatant was used for the detection of lipid peroxidation by Lipid Peroxidation MDA Assay Kit (#A003-1, Nanjing Jiancheng Bioengineering Institute, Nanjing, Jiangsu, China) according to the manufacturer’s instructions.

### Detection of iron ion concentration

1 × 10^5^ HL-60 and KG1a cells were seeded in the well of 24-well plates and treated with G-CSF or rhTPO. Next, iron ion concentration was determined using Iron Colorimetric Assay Kit (#E1042, Applygen, Beijing, China) following the manufacturer’s protocol. Briefly, cells were collected and homogenized. Subsequently, the supernatant of cells was collected by centrifuge. Then the supernatant was incubated with iron reducer for 30 min followed by the incubation with iron probe for 1 h. Finally, absorbance at 593 nm was immediately detected by Multiscan MK3 (Thermo Fisher Scientific).

### Quantitative reverse transcription-PCR (qRT-PCR)

Total RNAs from HL-60 and KG1a cells were extracted using TRIZOL (Invitrogen, USA). The first-strand cDNA was made by PrimeScript II 1st Strand cDNA Synthesis Kit (TaKaRa Biotechnology, Dalian, Liaoning, China) according to the manufacturer’s instructions. The amount of target RNA was normalized to the amount of internal control (GAPDH) and the results were given by 2^−△△Ct^ relative to the control sample. The qRT-PCR was performed by SYBR Green (Takara Biotechnology, China). The primer sequence was as follows: GPX4 forward: 5′-TCAGCAAGATCTGCGTGAAC-3′, reverse: 5′-GGGGCAGGTCCTTCTCTATC-3′; EP300 forward: 5′-CCTGAGTAGGGGCAACAAGAAGA-3′, reverse: 5′-ATGAGGCGGATCACAAAGAAGAC-3′; GAPDH forward: 5′-AACGGATTTGGTCGTATTGGG-3′, reverse: 5′-CCTGGAAGATGGTGATGGGAT-3′.

### Dual-luciferase reporter gene assay

*GPX4* gene promoter region was inserted into luciferase reporter gene vectors. After co-transfected with luciferase vectors and blank expression vector or EP300 expression vector, KG1a cells seeded into the 24-well plates were treated with or without rhTPO and subsequently subjected to luciferase activity determination by the Dual-Luciferase Reporter Assay System (Promega, Madison, WI, USA) at 48 h after transfection.

### Chromatin immunoprecipitation (ChIP)

In brief, KG1a cells were crosslinked by 1% formaldehyde for 15 min at RT and then stopped by Glycine. Next, KG1a cells were lysed by sonication to shear DNA. Subsequently, 25 mg DNA chromatin sample was adjusted to a total volume of 500 mL in 450 ml of the dilution buffer contained protease inhibitors. Chromatin samples were then incubated with 1 μg EP300 antibody (#ab275378, Abcam) or anti-rabbit IgG (Cell Signaling Technologies, Danvers, MA, USA) and incubated with protein A/G magnetic beads overnight at 4 °C with gentle rotation. After the overnight incubation, magnetic beads were collected by magnetic separation device (Thermo Fisher Scientific) and cleaned. Next, immunoprecipitated DNAs were eluted with 100 μL elution buffer contained Proteinase K at 62 °C for 2 h. Then DNAs were purified and dissolved in the elution buffer. Finally, chromatin DNAs were analyzed by PCR and qRT-PCR. Primers used were listed as followed: GPX4 promoter 1 forward: 5′-CTGGGCAACACAGCAAGA-3′, reverse: 5′-GGCCAGACAACCTGAGAATAC-3′; GPX4 promoter 2 forward: 5′- CATGCGCAGTCGCCAAC-3′, reverse: 5′-AGACGCGTCGGTGTTGAG-3′; GAPDH promoter forward: 5′-AAAAGCGGGGAGAAAGTAGG-3′, reverse: 5′-AAGAAGATGCGGCTGACTGT-3′.

### Statistical analysis

Quantitative data were present as mean ± standard deviation (SD) in this study. Statistical differences were analyzed using SPSS 20 software (SPSS Inc., Chicago, IL, USA). Samples and animals were randomly allocated to each experimental group and blinded to the investigator. Besides, estimation tests for sample size were not carried out in our animal studies. Moreover, no samples or animals were excluded from the analysis. In addition, the unpaired Student’s *t*-test was performed for the comparation between two groups, while statistics among multiple groups were analyzed by the post-hoc Tukey’s test following One way ANOVA. *P* < 0.05 was considered as statistically significant.

## Results

### Combination of G-CSF and rhTPO shows greater effect on suppressing leukemia cell proliferation and inducing its death than G-CSF or rhTPO alone

**Fig. 1:**
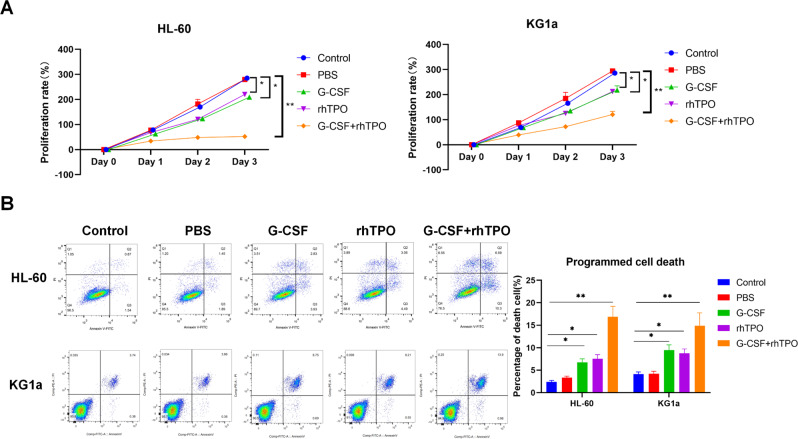
Combination of G-CSF and rhTPO shows greater effect on suppressing leukemia cell proliferation and inducing its death than G-CSF or rhTPO alone. **A** The proliferation rate of HL-60 cells or KG1a cells treated with combination of G-CSF and rhTPO, G-CSF or rhTPO. **B** Representative images of flow cytometric analysis for programed cell death in HL-60 cells and KG1a cells. The bar graph showed the quantification of death cell number in each group. *P < 0.05, **P < 0.01.

To identify the effect of combination of G-CSF and rhTPO on HL-60 and KG1a cell proliferation, CCK-8 assay was performed. Results found that combination of G-CSF and rhTPO, G-CSF alone and rhTPO alone could suppress HL-60 and KG1a cell proliferation, and combination of G-CSF and rhTPO showed greatest effect (Fig. 1A). Besides, HL-60 and KG1a cell proliferation was dramatically inhibited at 48 h (Day 2) after the treatment of combination of G-CSF and rhTPO (Fig. 1A). Thus, HL-60 and KG1a cells were treated by combination of G-CSF and rhTPO for 48 h in this study.

In addition, programed cell death of HL-60 and KG1a cells was detected using Annexin V and flow cytometric analysis. Results showed that combination of G-CSF and rhTPO induced programed cell death of HL-60 cells more effectively than G-CSF or rhTPO alone (Fig. 1B). Therefore, these results suggested that combination of G-CSF and rhTPO showed greater effect on suppressing leukemia cell proliferation and inducing leukemia cell death than G-CSF or rhTPO alone.

### Combination of G-CSF and rhTPO exerts greater effect on inhibiting leukemia growth than G-CSF or rhTPO alone in vitro

**Fig. 2:**
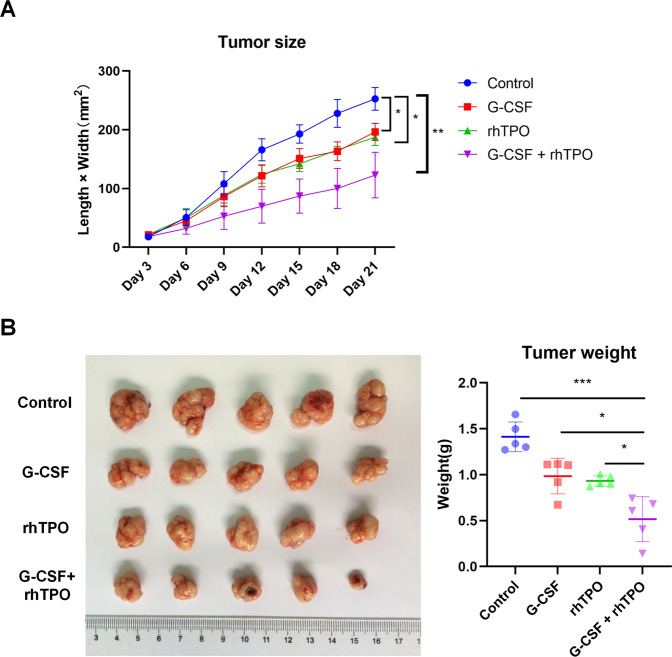
Combination of G-CSF and rhTPO exerts greater effect on inhibiting leukemia growth than G-CSF or rhTPO alone in vitro. **A** Volume of xenograft tumors formed in nude mice injected with KG1a cells and treated with combination of G-CSF and rhTPO, G-CSF or rhTPO. **B** Representative images of xenograft tumors formed in nude mice injected with KG1a cells and treated with combination of G-CSF and rhTPO, G-CSF or rhTPO. The bar graph showed weights of xenograft tumors. *P < 0.05, ***P < 0.001.

To further confirm the effect of combination of G-CSF and rhTPO on leukemia in vivo, a xenograft tumor mouse model was established by subcutaneously injecting KG1a cells into the right dorsal flanks of nude mice followed by tail vein injection of G-CSF and rhTPO. Results showed that tumors developed from KG1a cells in mice treated with combination of G-CSF and rhTPO, G-CSF alone and rhTPO alone were smaller and lighter than tumors in mice of control group (Fig. 2A–C). Besides, tumors developed from KG1a cells in mice treated with combination of G-CSF and rhTPO were smallest and lightest (Fig. 2A–C). Thus, these data indicated that combination of G-CSF and rhTPO exerted greater effect on inhibiting leukemia growth than G-CSF or rhTPO alone in vitro.

### G-CSF but not rhTPO triggers pyroptosis of leukemia cells

**Fig. 3:**
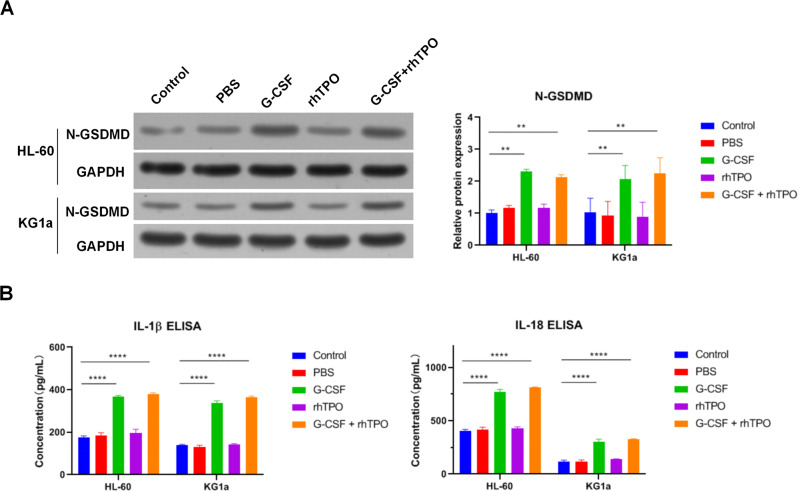
G-CSF but not rhTPO triggers pyroptosis of leukemia cells. **A** The protein level of N-GSDMD in HL-60 cells and KG1a cells treated with combination of G-CSF and rhTPO, G-CSF or rhTPO. **B** The levels of IL-1β and IL-18 in the cultured medium detected by ELISA. **P < 0.01, ****P < 0.0001.

Above results had indicated that G-CSF and rhTPO induced programed cell death of HL-60 and KG1a cells. Pyroptosis is an inflammatory programmed cell death and numerous inflammatory cytokines are involved in this progress [22, 23]. As G-CSF and rhTPO are related to inflammatory, the effect of G-CSF and rhTPO on pyroptosis was detected. Pyroptosis leads to Gasdermin D (GSDMD) cleavage and release of inflammatory cytokines such as interleukin-1β (IL-1β) and IL-18 [22, 24]. Therefore, cleaved GSDMD and released IL-1β and IL-18 are markers for pyroptosis. WB and ELISA results demonstrated that combination of G-CSF and rhTPO and G-CSF alone increased the level of cleaved GSDMD and released IL-1β and IL-18 in HL-60 and KG1a cells, whereas rhTPO alone had no effect on the cleavage of GSDMD and the release of IL-1β and IL-18 (Fig. 3A, B). Thus, these data indicated that G-CSF but not rhTPO triggered pyroptosis of leukemia cells.

### G-CSF induces pyroptosis through neutrophil elastase (ELANE) in leukemia cells

**Fig. 4:**
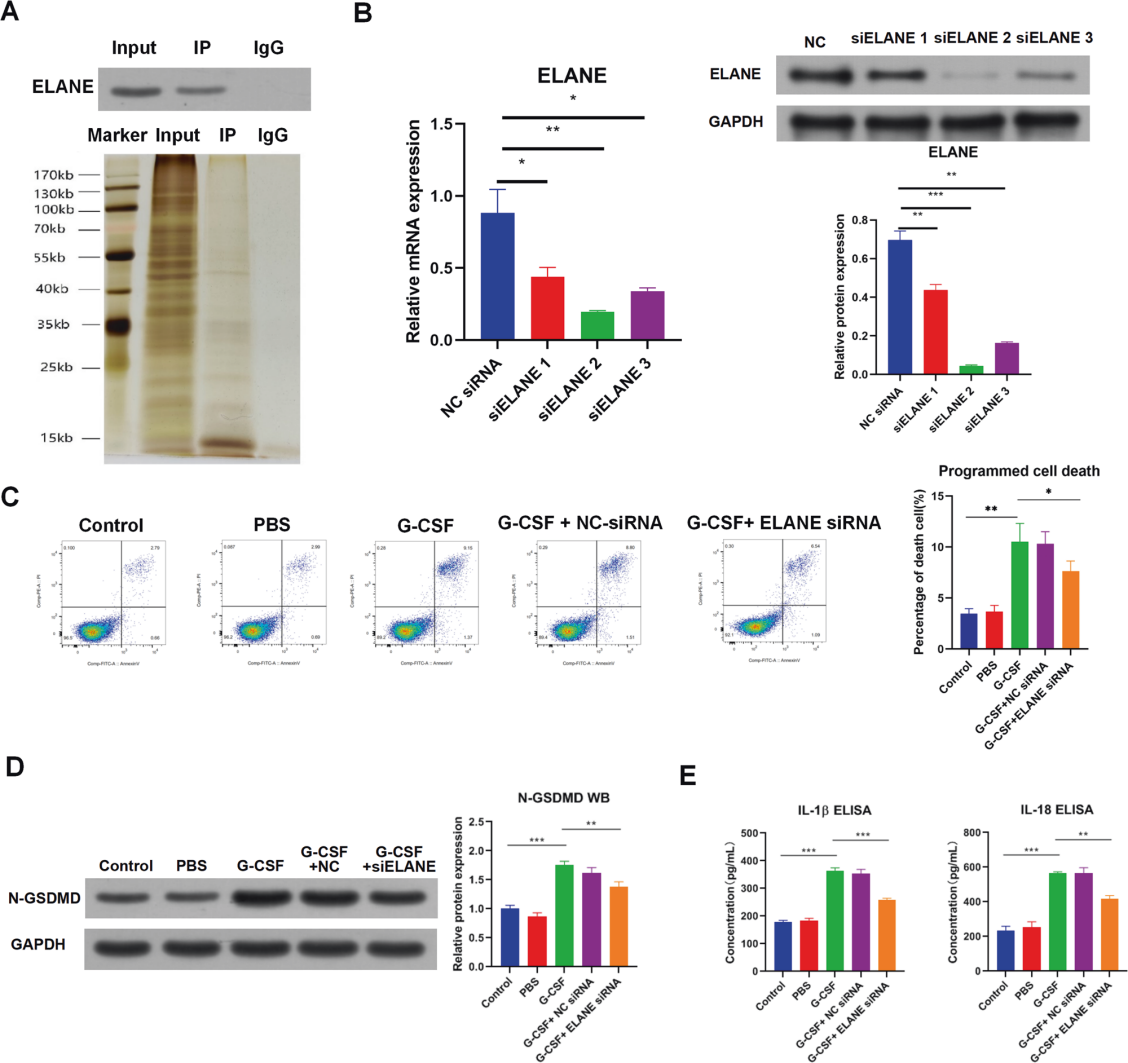
G-CSF induces pyroptosis through ELANE in leukemia cells. **A** Representative images of Co-IP using a G-CSF antibody in KG1a cells followed by detection of ELANE protein level by WB. Rabbit IgG was used as negative control. **B** The mRNA and protein level of ELANE in KG1a cells treated with ELANE siRNAs. **C** Representative images of flow cytometric analysis for programed cell death in KG1a cells treated with G-CSF or combination of G-CSF and ELANE siRNA. The bar graph showed the quantification of death cell number in each group. **D** The protein level of N-GSDMD in KG1a cells treated with G-CSF or combination of G-CSF and ELANE siRNA. **E** The levels of IL-1β and IL-18 in the cultured medium of KG1a cells treated with G-CSF or combination of G-CSF and ELANE siRNA detected by ELISA. NC: negative control. *P < 0.05, **P < 0.01, ***P < 0.001.

Next, the mechanism of G-CSF triggering pyroptosis of leukemia cells was explored. A previous study has revealed that G-CSF interacts with ELANE [25]. ELANE could induce pyroptosis through cleaving and activating GSDMD [26]. First, Co-IP was performed using anti-G-CSF to identify whether G-CSF associated with ELANE in leukemia cells. Results found that G-CSF interacted with ELANE in KG1a cells (Fig. 4A).

To further determine whether G-CSF induced pytoptosis of leukemia cells through ELANE, siRNA was utilized to silence ELANE expression in KG1a cells. Indeed, transfection of ELANE siRNA dramatically silenced ELANE mRNA and protein expression (Fig. 4B), and ELANE siRNA2 was utilized for subsequent experiments. Next, flow cytometric analysis indicated that silence of ELANE reversed the effect of G-CSF on programmed cell death of KG1a cells (Fig. 4C). Moreover, transfection of siRNA NC had no effect on programmed cell death of KG1a cells (Fig. 4C). Furthermore, WB results showed that silence of ELANE increased G-CSF-reduced cleaved GSDMD in KG1a cells (Fig. 4D). In addition, silence of ELANE abolished the effect of G-CSF on the release of IL-1β and IL-18 in KG1a cells (Fig. 4E). These results together suggested that G-CSF induced pyroptosis through ELANE in leukemia cells.

### RhTPO but not G-CSF induces ferroptosis of leukemia cells

**Fig. 5:**
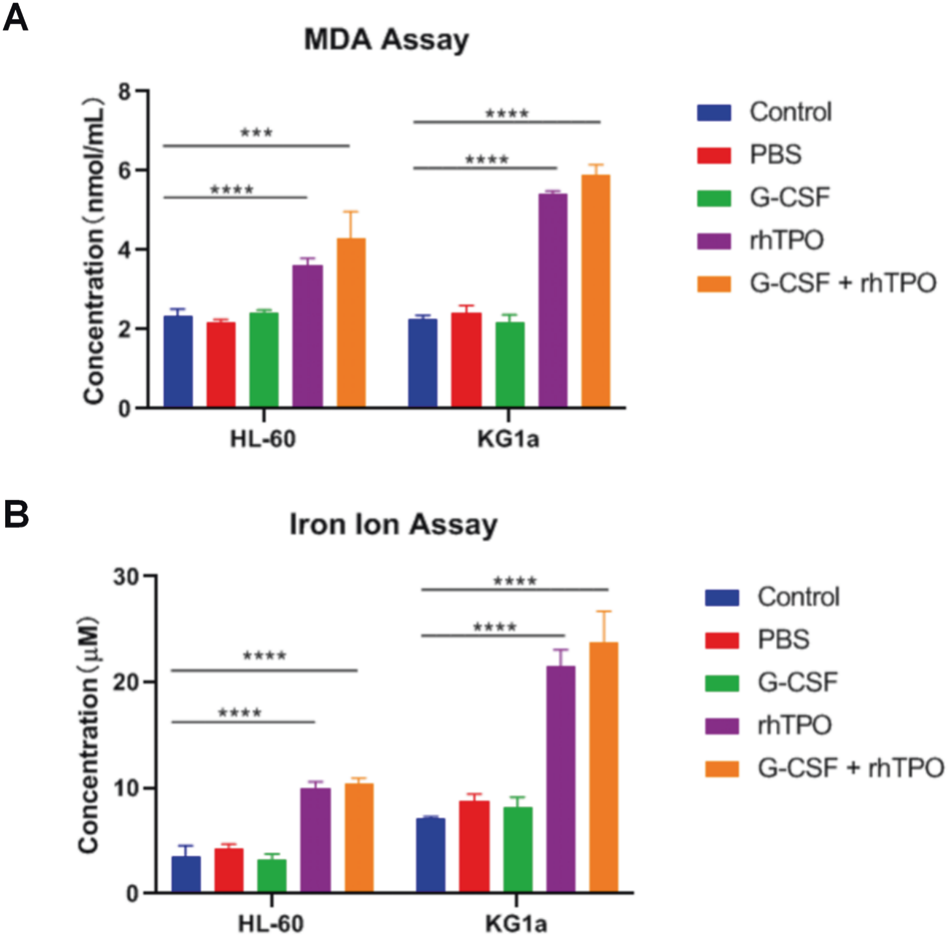
RhTPO but not G-CSF induces ferroptosis of leukemia cells. **A** The level of MDA in HL-60 cells or KG1a cells treated with combination of G-CSF and rhTPO, G-CSF or rhTPO. **B** The intracellular ferrous iron levels in HL-60 cells or KG1a cells treated with combination of G-CSF and rhTPO, G-CSF or rhTPO. ***P < 0.001, ****P < 0.0001.

Ferroptosis is a new identified type of programmed cell death and caused by accumulation of iron-dependent lipid peroxidation, which has been found in leukemia [27–29]. However, the roles of G-CSF and rhTPO in ferroptosis are largely unknown. Thus, markers for ferroptosis including MDA level and iron ion concentration were detected followed by the treatment of combination of G-CSF and rhTPO, G-CSF alone and rhTPO alone. Results revealed that combination of G-CSF and rhTPO and rhTPO alone elevated MDA level and iron ion concentration in HL-60 and KG1a cells, yet G-CSF alone had no effect on MDA level and iron ion concentration (Fig. 5A, B). Above data suggested that rhTPO but not G-CSF triggered ferroptosis of leukemia cells.

### RhTPO triggers ferroptosis by E1A binding protein P300 (EP300) in leukemia cells

**Fig. 6:**
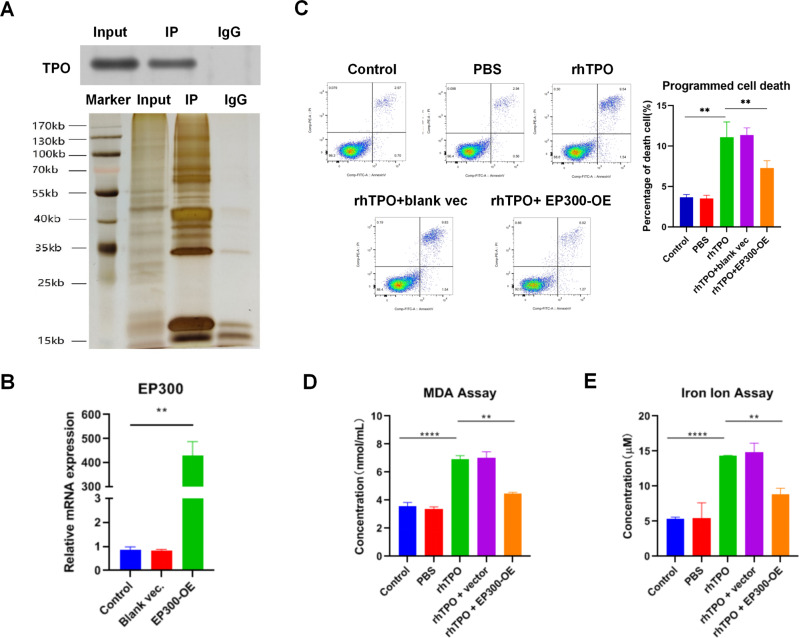
RhTPO triggers ferroptosis by E1A binding protein P300 (EP300) in leukemia cells. **A** Representative images of Co-IP using a EP300 antibody in KG1a cells followed by detection of TPO protein level by WB. Rabbit IgG was used as negative control. **B** The mRNA level of EP300 in KG1a cells transfected with EP300 expression vector. **C** Representative images of flow cytometric analysis for programed cell death in KG1a cells treated with rhTPO or combination of rhTPO and EP300 expression vector. The bar graph showed the quantification of death cell number in each group. **D** The level of MDA in KG1a cells treated with rhTPO or combination of rhTPO and EP300 expression vector. **E** The intracellular ferrous iron levels in KG1a cells treated with rhTPO or combination of rhTPO and EP300 expression vector. OE overexpression, vec expression vector. **P < 0.01, ****P < 0.0001.

Subsequently, the mechanism of rhTPO inducing ferroptosis of leukemia cells was illuminated. A previous study has demonstrated that TPO associates with EP300 [30], which could enhance *GPX4* gene transcription to inhibit ferroptosis [31]. Then Co-IP was performed using anti-TPO to determine whether TPO interacted with EP300 in leukemia cells. Results confirmed that TPO associates with EP300 in KG1a cells (Fig. 6A).

To further identify whether rhTPO induced ferroptosis of leukemia cells by EP300, EP300 was overexpressed by transfection of expression vector into KG1a cells. Transfection of EP300 expression vector significantly increased EP300 expression (Fig. 6B). Next, flow cytometric analysis revealed that EP300 overexpression neutralized the effect of rhTPO on programmed cell death of KG1a cells (Fig. 6C). Besides, transfection of blank expression vector had no effect on programmed cell death of KG1a cells (Fig. 6C). Moreover, EP300 overexpression abolished the effect of rhTPO on increasing MDA level and iron ion concentration in KG1a cells (Fig. 6D, E). These results indicated that rhTPO triggered ferroptosis by EP300 in leukemia cells.

### RhTPO suppresses GPX4 expression through blocking the interaction between EP300 and *GPX4* gene promoter via associating with EP300 in leukemia cells

**Fig. 7:**
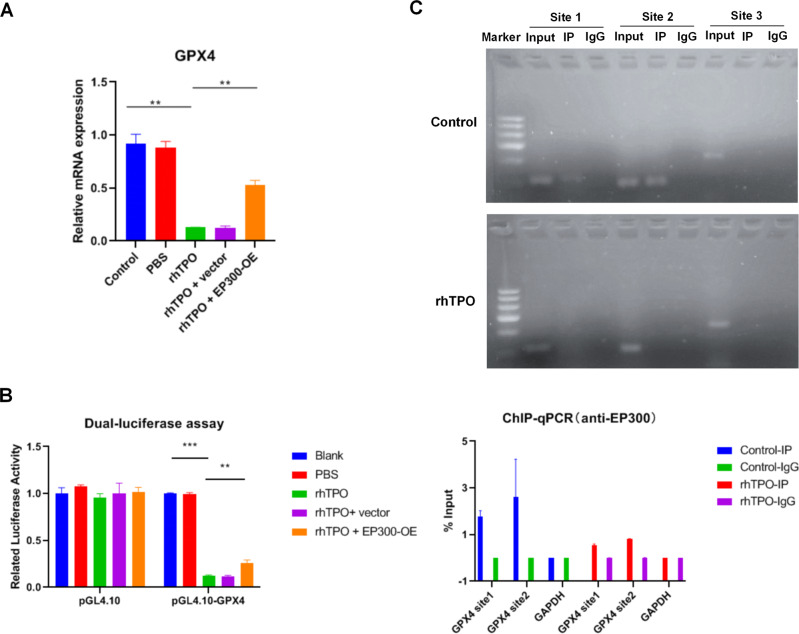
RhTPO suppresses GPX4 expression through blocking the interaction between EP300 and *GPX4* gene promoter via associating with EP300 in leukemia cells. **A** The mRNA level of EP300 in KG1a cells treated with rhTPO or combination of rhTPO and EP300 expression vector. **B** *GPX4* gene promoter activity analyzed by relative luciferase reporter activities in KG1a cells treated with rhTPO or combination of rhTPO and EP300 expression vector. **C** Immunoprecipitated chromatin associated with EP300 was analyzed using ChIP followed by PCR for the *GPX4* gene promoter in KG1a cells treated with or without rhTPO. The bar graph showed the quantification of EP300 occupancy on the *GPX4* gene promoter by qRT-PCR in KG1a cells treated with or without rhTPO. OE overexpression, vec expression vector. **P < 0.01, ***P < 0.001.

As EP300 regulates *GPX4* gene transcription to inhibit ferroptosis [31], the effect of rhTPO on *GPX4* gene transcription was detected. QRT-PCR analysis showed that rhTPO reduced GPX4 mRNA level whereas EP300 overexpression reversed the effect of rhTPO on GPX4 mRNA expression (Fig. 7A). Besides, dual-luciferase reporter gene assay revealed that the luciferase activity of KG1a cells transfected with reporter gene vectors containing *GPX4* gene promoter region was decreased by the treatment of rhTPO, whereas the effect of rhTPO was abolished by the transfection of EP300 expression vector (Fig. 7B). These results suggested that rhTPO suppressed *GPX4* gene transcription through EP300.

EP300 modifies *GPX4* gene transcription through binding with *GPX4* gene promoter [31]. Therefore, the effect of rhTPO on the association between EP300 and *GPX4* gene promoter was determined by ChIP. Results indicated that EP300 bound with *GPX4* gene promoter in KG1a cells (Fig. 7C). However, rhTPO inhibited the binding of EP300 with *GPX4* gene promoter (Fig. 7C). In addition, above results had indicated that rhTPO associated with EP300. Thus, rhTPO suppressed GPX4 expression through blocking the interaction between EP300 and *GPX4* gene promoter via associating with EP300 in leukemia cells.

## Discussion

This study revealed that combination of G-CSF and rhTPO showed greater effect on suppressing leukemia growth than G-CSF or rhTPO alone in vitro and in vivo. Mechanistically, G-CSF induced pyroptosis through ELANE in leukemia cells. Besides, rhTPO triggered ferroptosis by EP300 in leukemia cells. Moreover, rhTPO suppressed GPX4 expression to induce ferroptosis through blocking the interaction between EP300 and *GPX4* gene promoter via associating with EP300.

Numerous studies have indicated that G-CSF could induce apoptosis of leukemia cells. For instance, G-CSF induces apoptosis in leukemia cells through upregulating microRNA-146a [32]. Besides, G-CSF augments cytarabine and etoposide-triggered apoptosis in leukemia cells [33, 34]. Moreover, G-CSF triggers apoptosis in radiation-induced murine leukemia cell line C2M-A5 [35]. Except leukemia cells, G-CSF also induces apoptosis of B cells in bone marrow derived from healthy donors [36]. These studied have demonstrated that G-CSF could induce programmed cell death in leukemia cells and other cells. However, the role of G-CSF in pyroptosis remains unknown. Thus, our study revealed the effect of G-CSF on inducing pyroptosis for the first time.

The role of rhTPO or TPO on programmed cell death is controversial. For example, TPO prevents apoptosis of H9C2 cells against doxorubicin-induced cardiotoxicity [37]. Besides, TPO attenuates aplastic anemia serum-induced apoptosis in the mouse myeloid progenitor cells through STAT3/STAT5 pathway [38]. In addition, TPO reduces apoptosis of megakaryocytes [39]. By contrast, TPO induces apoptosis of porcine ovarian follicular cells [40]. More importantly, a recent study has indicated that rhTPO promotes apoptosis of HL-60 cells [41]. To date, the effect of rhTPO or TPO on ferroptosis is largely unknown. Therefore, the present study demonstrated the role of rhTPO in triggering ferroptosis for the first time.

In fact, G-CSF therapy attenuates ELANE mutation-caused neutropenia which might develop leukemia [25, 42]. However, the regulatory effect of G-CSF on ELANE has not been reported. G-CSF could modify the activation of target protein [43], so it might bind with ELANE to activate ELANE and subsequently induce pyroptosis in leukemia cells.

A previous study has demonstrated that EP300 interacts with cAMP response element‑binding protein (CREB) and facilitates the binding between CREB and *GPX4* gene promoter, and subsequently CREB enhances *GPX4* gene transcription to inhibit ferroptosis [31]. This study found that rhTPO suppressed GPX4 expression to induce ferroptosis through blocking the interaction between EP300 and *GPX4* gene promoter via associating with EP300. Thus, rhTPO might suppress the association between EP300 and CREB by competitive binding EP300 in leukemia cells.

Numerous types of programmed cell death are simultaneously involved in the progress of human disease. Therefore, the promising therapeutic targets, drugs or combined therapies usually regulate more than one type of programmed cell death concurrently. For example, mixed lineage kinase 3-induced pyroptosis and ferroptosis of cardiomyocytes contributes to myocardial fibrosis [44]. Besides, tumor-specific antigens and neoantigens are ideal targets to trigger pyroptosis and ferroptosis in cancer cells simultaneously [45]. In addition, histone deacetylase inhibitor quisinostat induces apoptosis, pyroptosis, and ferroptosis in tongue cancer cells at the same time [46]. Therefore, pyroptosis/ferroptosis dual-inductive combinational anti-cancer therapy could improve curative effects [47], which is why combination regimen of G-CSF and rhTPO shows greater effects than G-CSF or rhTPO alone.

## Conclusion

In summary, this study demonstrated that combination of G-CSF and rhTPO showed greater effect on suppressing leukemia growth than G-CSF or rhTPO alone in vitro and in vivo. Mechanistically, G-CSF induced pyroptosis through ELANE in leukemia cells. Besides, rhTPO triggered ferroptosis by EP300 in leukemia cells. Moreover, rhTPO suppressed GPX4 expression to induce ferroptosis through blocking the interaction between EP300 and *GPX4* gene promoter via associating with EP300. These results provided a theoretical basis for combination regimen of G-CSF and rhTPO treating leukemia and potential therapeutic targets for leukemia.

## Availability of supporting data

The datasets generated during and/or analyzed during the current study are available from the corresponding author on reasonable request.
